# Risk factor analysis of iodinated contrast medium-related hypersensitivity reactions

**DOI:** 10.1186/s13244-025-02099-y

**Published:** 2025-10-10

**Authors:** Lukas Beiner, Ingrid Boehm

**Affiliations:** 1 MediZentrum Landhaus Steffisburg AG, Steffisburg, Switzerland; 2 Department of Diagnostic, Interventional, and Pediatric Radiology, University Hospital of Bern, Inselspital, University of Bern, Bern, Switzerland

**Keywords:** Adverse drug reaction, Contrast media, Hypersensitivity, Patient safety, Risk management

## Abstract

**Abstract:**

Hypersensitivity reactions caused by iodinated contrast media (ICM) are by definition type B adverse reactions and therefore, they are not predictable. To partially limit this uncertainty, since the 1980s, risk factors have been defined and published. Currently, there are so many risks that any patient undergoing a contrast-enhanced imaging examination would have at least one risk. This is not helpful and instead leads to uncertainty again. From both studies and clinical experience, we know that only a small percentage of patients develop hypersensitivity reactions after ICM administration. Therefore, we subjected the risks published to a critical analysis. Based on 126 publications, we identified 43 risks, which were divided into three categories (patient-related, contrast agent-related and management-related risks). We have also mentioned the appropriate management for each risk. After critical assessment, the risk status remains with a history of an ICM-hypersensitivity reaction, acute allergic symptoms, the culprit ICM and documentation errors (e.g., if an iodine allergy is mentioned, a latex allergy is incorrectly suspected as an ICM-allergy or the wrong trigger is documented). In addition, we found that several risks have been named differently, although they cover the same risk situation (e.g., chronic/severe disease and frequent ICM applications). Furthermore, for several of the published risks, no mitigation measures are available. Taken together, of the large number of published risks, only those with risk status should be used clinically in the future. Known risk factors do not influence the nature of type B reactions.

**Critical relevance statement** For patient safety, it would be advisable in the future to consider the following three risks: a history of an ICM-hypersensitivity reaction, acute allergic symptoms and documentation errors (e.g., if an iodine allergy is mentioned, a latex allergy is incorrectly suspected as an ICM-allergy or the wrong trigger is documented).

**Key Points:**

- Risk factors are intended to limit the unpredictability of ICM hypersensitivity reactions.
- Currently, risks are nonspecific and ultimately apply to all patients.
- We found that identical factors are published under different names.
- Four risks are relevant: history of ICM-HSR, acute allergy, the culprit ICM and documentation/management errors.

**Graphical Abstract:**

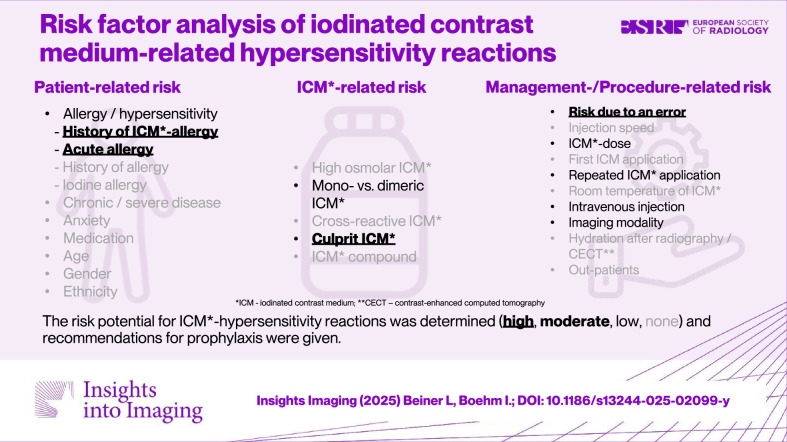

## Introduction

Hypersensitivity reactions (HSR) following the application of iodinated contrast media (ICM) are still challenging in radiological routine practice. These reactions are, *per definitionem*, so-called type B reactions. This means they are independent of the pharmacology of the applied drug and not predictable (in contrast, type A reactions are dependent on the pharmacology and predictable [see section “Definition of type A and B reactions”]) [1, 2]. This prompted clinicians and scientists around the world to search for risk factors that could limit unpredictability. During the past, many (potential) risk factors have been published [3–7]. The list of risk factors varies greatly from paper to paper. In addition, there are sometimes so many risks that ultimately almost all patients who receive a contrast medium as part of an imaging examination would be affected. Therefore, papers presenting risk factors cause confusion [8] and defeat the purpose of improving the safety of CM administration. In a survey, only 13% of the participants were able to identify risk factors [9]. Now, there seem to be too many risk factors. Therefore, there are critical discussions about the usefulness of the risk factors in the literature [7, 8, 10].

In order to enable effective management, radiologists should know clinically relevant risk factors. On the other hand, prophylactic measures for pseudo-risks are time- and personnel-intensive, but without clinical benefit. Both scenarios, over- and under-treatment, can harm the patient. Risk factors of ICM-induced anaphylaxis are not clearly defined [11]. Therefore, the goal of the present paper is threefold: to categorize published risks, to analyze their risk potential, and to present the recommended prophylaxis. Moreover, we also aimed to identify new, previously unknown risk factors. We focus on hypersensitivity (type B) reactions, and also mention type A reactions for a better understanding or for differentiation when necessary for completeness.

## Materials and methods

### Literature search

We performed a careful literature search by using the PubMed online database and the following keywords in different combinations: “iodinated contrast media,” “hypersensitivity,” “allergy,” “allergy-like reaction,” and “risk factor.” We also searched in detail for keywords such as “age,” “gender,” “history of ICM hypersensitivity,” for example. In addition, we searched literature from other sources, such as the reference lists of identified publications.

We included review papers, original papers, case reports, adverse drug reactions (ADR) induced by ICM, and paper languages in either English or German that were freely available online. We excluded papers that deal with contrast-induced acute kidney injury, focus on gadolinium-based and ultrasound contrast agents and/or are presented in other languages.

### Risk definition and classification

We define risk for the acquisition of ICM-HSR as a special condition (e.g., previous history of ICM hypersensitivity, acute allergic asthma) or insufficient documentation of such a condition in the past in individual patients. The aim of identifying risks is to carry out adequate risk management, i.e., to develop strategies to minimize or completely control the risks. In the first step, we classified the risks in terms of the cause (patient-, ICM-, and procedure-/management-related risks [12]). In the second step, we specifically listed the risks. Then we analyzed their risk status as high, medium, low or non-existent. Finally, we provide recommendations for individual management based on literature data and our expertise.

### Definition of type A and B reactions

According to Rawlins and Thompson, type A reactions are predictable, common, and related to the pharmacological properties of the drug. In contrast, type B reactions are hypersensitivity reactions (allergy (allergy test positive, either intradermal or patch test [3, 6]) and non-allergy) that are unpredictable, uncommon, and usually not related to the pharmacological properties of the drug [1, 2]. We mainly focus on type B reactions, but also mention type A reactions when it helps to understand the whole context for completeness. The term “adverse reaction” covers all reaction types (i.e., type A and B reactions). Where necessary, we explain the term in the text.

## Results and practical recommendations

### Study overview

**Fig. 1:**
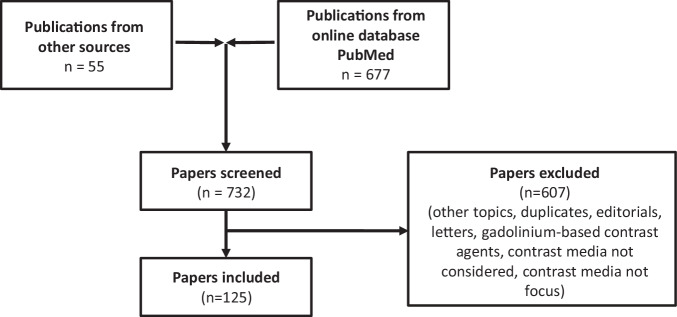
Flowchart shows the selection of literature based on publications from the literature

We found 732 papers, excluded 607 of them, and analyzed risk factors in 125 papers (Fig. 1). We identified 43 risk factors (Table 1). The most prominent risk in the literature is a history of hypersensitivity/allergy to ICM and a history of another allergy, followed by female gender. Potential risks vary greatly from paper to paper. Consequently, there is no uniform group of potential risks in the literature. We identified the following three main groups: patient-, ICM- and procedure-/management-related risks, which we divided into several subgroups. Risks due to an error (see section “Risks due to an error”) are presented for the first time.

**Table 1:** Risk factors, risk potential and recommended management of contrast media hypersensitivity reactions sorted into three risk categories: patient-related, ICM-related and management-related risks

Risk factor	Risk potential	Recommended management
Patient-related risks		
Acute allergy	Moderate to high	Elective ICM-enhanced CT examinations should be performed when patients do not suffer from acute symptoms, and emergency contrast-enhanced examinations should be done with special pre-care (possibly stand-by of an emergency doc)
Pollen seasons (= acute allergy)		
History of hypersensitivity/allergy to ICM	Moderate to high, depending on the severity and timing of the last ICM-HSR	Perform an individual management. Mild reactions ➜ omit the culprit ICM for future contrast-enhanced imaging procedures. Moderate reactions ➜ use allergy skin test results to decide which contrast medium is tolerated best. Serious reactions ➜ consider emergency medical standby or switching to another imaging modality in addition to allergic skin test results.
History of iodine allergy	Moderate	Omit the term “iodine allergy”
Family history of ICM hypersensitivity	Unknown to none	None
History of allergy (drug allergy, atopy, asthma bronchiale, allergic rhinitis, food allergy)	Low to none	None
Serious/chronic disease	None	None
Patient’s anxiety	Moderate to high	Radiologists should develop a feeling for anxious patients and respond carefully to their concerns. Avoid anything that could frighten the patient.
The way of talking to patients		
Medication	None	None
Gender	None	None or individual contrast dose adjustment in women
Age	None	None
Ethnic background	Unknown	None
ICM-related risks		
ICM compound (e.g., iopromide, iodixanol)	None	Apply non-culprit ICM
Culprit ICM	Low to high	Document the culprit ICM following an adverse reaction and avoid it when performing ICM-based procedures in the future. If contrast is necessary, apply a non-culprit ICM.
Cross-reacting ICM	None to low	Apply non-culprit ICM
Osmolality (HOCM > LOCM > IsoOCM)	Moderate to high for type A reactions; none to low for type B reactions	Apply low- or iso-osmolar ICM
Mono- versus dimeric ICM	Moderate to high for dimeric compounds to induce non-immediate reactions	Apply monomeric ICM in patients with dimeric ICM hypersensitivity and vice versa
Management-/procedure-related risks		
Incorrect assumption regarding the culprit ICM	High	Exact documentation of the culprit ICM
ICM is incorrectly assumed to be the culprit agent	Moderate to high	Search for the individual culprit factor
Incorrect documentation and incorrect diagnosis, such as “iodine allergy”	Moderate to high	Omit the term “iodine allergy”
Patient mix-up	Moderate to high	Always make sure you are examining the right patient
Over- and underestimation of risks	Moderate to high	Carry out a risk-benefit assumption in each case
Outpatient	Low	Improve documentation of ICM-related HSRs
High injection speed	Moderate for type A reactions	In patients who have responded to a high injection rate with corresponding symptoms, as prophylaxis, the lowest possible rate should be used. Injection speed does not predispose to ICM-HSRs.
ICM dose	Depends on the amount of ICM applied and is greatest at the medium dose administered as part of a CECT	In patients who react to standard doses but do not react to low-dose regimens, the latter should be applied if possible
First ICM injection	None	None
Repeated ICM injection or history of prior ICM exposure	Moderate	None
ICM at room temperature	Low for type A reactions	Pre-warming the ICM to 37 °C when necessary
Intravenous ICM injection	Low to medium	None
Imaging modality (CECT)	None	None

*ACE* angiotensin-converting enzyme, *BMI* body mass index, *CECT* contrast-enhanced computed tomography, *HOCM* high-osmolal contrast medium, *ICM* iodinated contrast media, *IR* immediate reaction, *LOCM* low-osmolal contrast medium, *NIR* non-immediate reaction

The first papers that focused on various risk factors for ICM-HSR were from 1980 [13, 14] (with a special exception from the 1970s, see section “Anxiety”).

Odds ratios (OR) were reported in only a subset of the included references, specifically, in 28 studies (for detailed data, see Supplement 1 and 2).

This work is the first to critically analyze possible risks for an ICM-HSR for their clinical relevance. For enhanced clinical orientation, we grouped the identified risks as patient-, ICM-, and procedure-/management-related risks [12], determined the risk status (high, medium, low, or none) and recommended appropriate management.

### Problems with nomenclature

Papers on risk factors should add useful information on adverse CM-induced reactions. Therefore, it is not only necessary to mention the kind of risk but also the expected reaction. For example, the history of ICM allergy is a risk for an ICM allergy.

In this context, it is important to differentiate between type A and type B reactions [1]. Already about 30 years ago, it was pointed out that there are predictable and unpredictable reactions [15]. However, terms such as “allergy-like reaction” or “physiological reaction” are preferred in the literature. These terms should be avoided because they are imprecise and confusing [16].

For example, describing patients with a history of “allergic reactions” as being at increased risk for a subsequent “physiological reaction” [7] is a misunderstanding due to the incorrect use of technical terms.

We also found differently named risks that mean the same risk, such as chronic disease and frequent ICM applications (Table 2).

**Table 2:** Identical risk factors with different names are marked as ‘x,’ and identical risks with identical names are marked as ‘o’

	Acute allergy	Pollen season	History of ICM-HSR	Chronic disease	Medication	Female gender	ICM compound	Culprit ICM	Outpatient	Insufficient document.	ICM dose	Repeated ICM inject.	i.v. ICM	CT
Acute allergy	o	x				x								
Pollen season	x	o				x								
History of ICM-HSR			o					x						
Chronic disease				o	x							x		
Medication				x	o									
Female gender	x	x				o								
ICM compound							o					x		
Culprit ICM			x					o						
Outpatient									o	x				
Insufficient documentation									x	o				
ICM dose											o		x	x
Repeated ICM injection				x	x							o		
i.v. ICM											x		o	x
CT											x		x	o

### Patient-related risks

#### Acute allergy

Patients with acute or poorly controlled allergy (e.g., asthma, drug/food allergy, allergic rhinitis) are at increased risk for the acquisition of ICM hypersensitivity. Based on the literature, it is sometimes difficult to decide whether acute symptoms of allergic diseases or merely anamnestic information about a corresponding allergy is reported [10, 11, 17–36].

The immunological situation in the organism may be responsible for this observation. An activated immune system apparently reacts more easily to the application of a contrast agent than a non-activated one. Therefore, pollen season is a risk for patients with such an allergy [4, 5, 36–40]. In addition, there are also papers that show no significant seasonality trend observed [7, 41]. This shows that the respective composition of the patient population is very different and influences the results. Moreover, the nomenclature is inconsistently used among many papers. Furthermore, what investigators exactly mean by “allergy” is rarely defined clearly [42].

Risk status: moderate to high.

Management: Elective ICM-enhanced CT examinations should be performed when patients do not suffer from acute symptoms, and emergency contrast-enhanced examinations should be done with special pre-care (possibly stand-by of an emergency doc).

#### History of previous hypersensitivity/allergy

*History of ICM hypersensitivity*. A history of ICM-HSR is the most significant risk factor for ICM hypersensitivity, as supported by numerous studies [5, 7, 8, 10–12, 18–24, 26–32, 34–37, 39–66]. Multivariate regression analysis has shown this high risk (OR = 40.693; *p* < 0.001 up to OR = 198.8) [26, 53].

However, it is largely unknown which specific reactions in a patient’s history predispose to which reactions. Patients with immediate reactions in their history are at risk for similar future immediate reactions, and those with non-immediate (delayed) reactions are at risk for a non-immediate reaction upon re-exposure to the culprit ICM [19]. Immediate reactions do not predispose for non-immediate reactions, and vice versa. The different pathophysiological mechanisms between these reaction types are responsible for this fact. Biphasic reactions, which involve both types, are an exception [67, 68]. In general, type B reactions predispose for type B only. The same is true for type A reactions [19]. Previous type A reactions do not predispose for type B reactions, and vice versa. Type A and B reactions have different pathological mechanisms, so a crossover between them is not possible. Therefore, the described situation that patients with a history of ICM-HSR had a higher likelihood of physiologic reaction [7] is not possible. There must be a mix-up. Since some symptoms can occur in both type A and type B reactions, differentiation is not always possible.

Hypersensitivity reactions to GBCA are not a risk for ICM reactions, and vice versa (the results of Ahn et al [69]—with OR as high as 4.6—are likely due to an acute allergy (see section “Acute allergy”) and indicate that we should not overestimate OR). In this context, we should be aware of an accurate documentation of adverse reactions (exact name of the culprit ICM, date of the reaction, clinical symptoms [70]), which is crucial for safe future ICM applications [6]. Insufficient documentation, imprecise terms and misdiagnosis of previous reactions are significant issues in radiology and can lead to further ADR [6, 11]. If the culprit ICM is known, it should be avoided in future procedures. There is no need to pay attention to cross-reactivity (see below) [71].

Risk status: moderate to high, depending on the severity and timing of the last ICM-HSR.

Management: perform an individual management.

Mild reactions ➜ omit the culprit ICM for future contrast-enhanced imaging procedures.

Moderate reactions ➜ use allergy skin test results to decide which contrast medium is tolerated best.

Serious reactions ➜ consider emergency medical standby or switching to another imaging modality in addition to allergic skin test results.

*History of “iodine allergy”*. So-called “iodine allergy” is also listed as a risk in the literature [28, 72, 73] (see section “Risks due to an error”). One should be aware that “iodine allergy” (the term covers allergy to shellfish/seafruits, ICM, amiodarone, and iodine-containing local disinfectants (e.g., povidone-iodine); for example, povidone allergy is an allergic skin reaction and has no relation with HSR to ICM) does not exist. Although this has been published previously [74, 75], both patients and doctors still use this inexact term. Formerly, it has been suspected that iodine is responsible for ICM-HSR [5]. Now it is clear that this is not the case. The omission of the culprit ICM and the application of a non-culprit compound is a safe prophylaxis for patients at risk [76]. Despite this fact, many radiologists still believe that it does not matter which ICM is administered. Therefore, intense regular training is necessary.

Risk status: moderate.

Management: omit the term “iodine allergy.”

*Family history of ICM hypersensitivity*. Family history of ICM-related HSR as risk is a special case that is rarely mentioned [50, 53]. Due to its rarity, an assessment is not possible. Additionally, one paper found that family history does not pose a risk [19].

Risk status: unknown to none.

Management: none.

*History of another allergy*. A history of allergy (e.g., atopy, asthma, chronic urticaria, drug/food allergies) is frequently cited as a risk factor for ICM-HSR [5, 7, 8, 10,12–14, 18, 20–22, 24, 26–30, 32–37, 39–44, 46–54, 56, 57,59, 61–66, 77–81]. As early as in 1980, Kalimo and colleagues found allergy history to be a statistically significant risk, but they also noted that many patients without ICM reactions also had positive allergy histories, casting doubt on its usefulness as a risk factor [14]. This point still applies. It is noteworthy that allergy per se is no risk factor for the acquisition of an ICM-HSR. Thus, it is more relevant to ask about current acute allergy symptoms rather than past allergies (see section “Acute allergy”). Several papers and guidelines list allergy history or atopy as a risk due to uncritical repetition of prior findings, inflating its significance [10]. In this way, factors that do not represent real risks are magnified.

Risk status: low to none.

Management: none.

#### Severe/chronic disease or condition

Patients with severe/chronic disease are more likely to undergo regularly contrast-enhanced CT scans than those without such complaints. The more often a patient receives an ICM, the greater the risk of acquiring contrast-hypersensitivity. Repeated exposures to ICM increase the risk of HSR [26, 57]. This appears to be the reason why the following factors are mentioned as risks:

- oncological diseases [26, 31, 39, 46, 56];
- systemic lupus erythematosus [24, 27, 37, 47, 48, 59, 82–84];
- renal complaints [5, 8, 18, 20, 27, 30, 31, 33–37, 39, 44, 51, 57, 62, 64, 85] possibly due to the longer persistence in the organism;
- increased body mass index (BMI) [7, 8, 34, 86];
- a variety of other diseases such as anemia, diabetes mellitus, gout, hepatic disease, cardiovascular disease (e.g., coronary artery disease, hypertension), and pulmonary disease [8, 10, 12, 19, 22, 26, 31–33, 36, 42, 44, 46, 80, 87, 88]. Even mastocytosis, which was previously considered a risk [10, 27, 28, 48, 49, 59, 89–95], does not have this status [96].

Risk status: none.

Management: none.

#### Anxiety

Even though anxiety is an important topic, only a few publications mention it [19, 28, 39, 42, 44, 97]. Patients with anxiety, first described by Lalli in 1974 [98], are more likely to develop an acute adverse reaction (i.e., type A reaction) to iodinated contrast media. Anxiety increases the risk of such acute reactions in patients undergoing contrast-enhanced computed tomography (CECT), suggesting the clinical importance of screening for anxiety before imaging examinations [97]. Although the study did not differentiate between type A and B reactions, it suggests that anxiety aggravates type A reactions (e.g., vasovagal reactions).

Fear can stem from various sources, including ICM injection (syringophobia), imaging results or general anxiety [99]. Recognizing anxiety allows the radiologist to intervene appropriately, such as by offering anxiolytic medication. However, when fear mimics other reactions like anaphylaxis, it can lead to incorrect treatment or prophylaxis.

The way radiologists communicate with patients is also crucial in managing anxiety. Studies show that informed consent can reduce anxiety, while excessive details can increase it [100–102].

Risk status: none for type B reactions.

Management: radiologists should develop a feeling for anxious patients and respond carefully to their concerns. Avoid anything that could frighten the patient. Possibly apply anxiolytic drugs.

#### Medication-related risk

Interactions between contrast media and other drugs are largely unknown. Researching them is difficult, among other things, because there are numerous drugs and drug combinations. The patient’s individual genetic constellation could also play a modifying role.

In the past, several drugs have been reported to increase the risk of a CM-HSR [28, 30, 31, 33, 39, 42, 43, 51, 59, 61, 88, 103–108]. The most important of such compounds are:

- beta-blockers [30, 31, 33, 39, 43, 59, 88, 105] and radiologist should be aware that patients taking this kind of medication (particular caution is necessary in patients with a history of ICM hypersensitivity), because beta-blockers do not increase the frequency/severity of HSR, but they may suppress initial symptoms of anaphylactic reactions, so that necessary treatment starts delayed and severe life-threatening situations can occur [109, 110];
- interleukin-2 (IL-2) [6, 28, 31, 33, 39, 42, 46, 51, 59, 61, 103, 107, 108, 111], although a recent paper suggests a possible causality [111], the current data are not suitable for considering a causal connection as likely [6, 46];
- angiotensin-converting enzyme (ACE) inhibitors can induce undesirable reactions depending on and independently of the contrast agent [31, 112];
- taxanes were suspected of being risk factors [104], but this is unlikely because the percentage of ADRs is also found in the general population [113];
- anti-CTLA4 antibodies were also suspected as risks [106], but the data are not sufficient.

In addition, we found an article in which concomitant drugs served as risk without specification [40].

Risk status: none.

Management: none.

#### Gender

Numerous papers list female gender as risk for the acquisition of a CM-hypersensitivity [5, 7, 8, 10, 17, 21–23, 26, 28, 29, 31, 33–35, 37, 38, 40, 42, 51, 59, 60, 65, 86, 114–123]. Women were also more commonly affected by delayed reactions [124] that could not be confirmed by others [125].

Females suffer more often from allergic diseases than males [42]. This could be the reason why women are more likely to acquire ICM hypersensitivities. There is another possible cause. Recently, Becker et al found that the contrast agent volume is regularly overdosed in women [126] (knowing that most centers now use weight-based dosing). The increased CM-dose alone or together with other (yet unknown) factors could be responsible for the increased risk. Thus, an individual dose adjustment should reduce the incidence of HSR in women as well as healthcare costs, the carbon footprint for drugs, and the contamination of drinking water with contrast agents [126–129]. Finally, one should take into account that women are more health-conscious than men are, and consult a doctor more often [130].

Moreover, there are also studies that did not confirm females at risk [46], in which the gender ratio is balanced [58, 63, 66, 80, 87, 131], or in which mainly men were affected by ICM-HSR [80, 122]. Ultimately, the role of gender in contrast-HSR is yet unclear.

Risk status: none.

Management: none or individual contrast dose adjustment in women.

#### Age

Age is an often-mentioned risk factor for the acquisition of CM-HSR [7, 8, 10, 19, 21, 22, 26, 29, 31–34, 38–40, 46, 52, 58, 60, 64, 66, 86, 87, 104, 114, 118, 132–134]. However, the age range information varies from publication to publication. While some authors place the risk in very young patients (< 20 years) [33, 34, 39], others see the risk in young adults (20–40 years) [7, 8, 21, 22, 29, 32–34, 52, 58, 60, 64, 66, 87, 118, 132, 134], mature adults (41–60 years) [7, 8, 10, 21, 22, 26, 29, 32, 38, 46, 86, 104, 114, 118, 133, 134] or in elderly patients (> 61 years) [10, 33, 39, 46, 60, 86, 104, 134] (Table 1). In summary, the literature shows the entire spectrum of ages as a potential risk. This means that age plays no role as a risk [58].

Risk status: none.

Management: none.

#### Ethnicity

Some authors mention ethnic origin as a risk [7, 13, 17, 21, 23, 42, 59, 116]. Some of them found a higher likelihood of HSR in non-white patients compared to white patients [7, 13, 21], other studies showed the opposite outcome [17, 116], and still others found no association between ethnicity and risk [23]. The data is inconsistent, making it difficult to classify ethnicity as a clear risk factor.

Risk status: unclear.

Management: none.

### ICM-related risk

#### ICM compound

The assumption of whether certain contrast agents pose as risk for ICM-HSR is regularly asked by patients, physicians and radiologists as well as raised in the literature [5, 7, 18, 26, 58, 114, 115, 118, 122, 132, 134, 135]. For example, iopromide was suspected as risk for acute reactions or special symptoms [5, 7, 118, 125]. In another study, iomeprol seemed to be the major contrast agent inducing severe immediate reactions, and iodixanol seemed to be responsible for non-immediate reactions (16% NIHR vs 3% IHR; *p* < 0.001) [10]. While others found that iobitridol was responsible for most HSR [56]. The authors explained this on the frequent use of iobitridol at their institute. This will probably also be the case with the other studies. The more frequently a contrast medium is used, the more likely HSR are to be expected. No contrast agent molecule represents a potential risk, which arises through the reactivity of the human organism.

Risk status: none.

Management: apply non-culprit ICM.

#### Culprit ICM

Initially, iodine was thought to be responsible for allergic reactions. Therefore, the principle applied: if one ICM is not tolerated, all other ICM are also not tolerated. Consequently, in patients with ICM hypersensitivity, it did not matter which ICM they received. Even the ICM that induced the initial reaction was reapplied. Since this procedure posed a high risk for the affected patient, an attempt was made to minimize it by administering anti-allergic premedication. Now we know that iodine is not responsible for the allergy [3, 6, 31, 33, 42, 48–51, 59, 74, 75]. This means that patients respond to individual ICM only. Re-administration of the culprit ICM leads to an allergic reaction again in 27.7% of cases [76]. With premedication, the risk is 17.3%. The latter are so-called breakthrough reactions [45]. In 1990, Katayama and colleagues for the first time showed that switching to a non-culprit CM reduces the incidence of adverse reactions (type A and B reactions) [87]. In their study, the authors switched from high to low-osmolar ICM (LOCM). Now, we know that switching to another ICM also works within the LOCM group [3, 31, 48, 71, 72, 76] (see section “Cross-reactive ICM”).

Risk status: low to high.

Management: document the culprit ICM following an adverse reaction and avoid it when performing ICM-based procedures in the future. If contrast is necessary, apply a non-culprit ICM.

#### Cross-reactive ICM

Currently, the application of a non-culprit contrast agent is established radiological practice [76, 136]. In this context, the question arises as to which alternative contrast agent is safest. Although the chemical structures of ICM molecules are all very similar, there are minimal differences. Based on these differences, Schmidt et al distinguished eight groups of ICM [71]. Theoretically, cross-reactivity can occur within such a group. Consequently, it seems logical to select the alternative ICM from a group that does not belong to the culprit one to avoid cross-reactivity [49, 72, 81, 125].

However, it turns out that the risks within and outside these chemical ICM-groups do not meet expectations (although individual data give a different impression). This means there need not be any reactivity within the chemical group. In other words, each patient appears to have an individual reaction pattern, without the chemical groups of the ICM seeming to play a significant role [71]. Consequently, radiologists do not need to know the chemical groups, but, if necessary, should send the patient for an allergy test to determine the individual reaction profile.

Risk status: none to low.

Management: apply non-culprit ICM.

#### High-osmolar ICM

Currently, high-osmolar ICM (HOCM) has been nearly completely replaced by low-osmolar ICM (LOCM). There are only a few indications for HOCM, such as gastrointestinal imaging (non-vascular, i.e., oral application). Nevertheless, we would like to mention HOCM here because there are still large gaps in knowledge regarding the risks. Therefore, with this chapter, we would like to contribute to the general understanding of hypersensitivity reactions.

HOCM were associated with a higher risk of adverse reactions compared to low- or iso-osmolar ICM, which are now commonly used due to their better tolerability [8, 17, 36, 44, 54, 87]. Without pre-treatment, patients with a history of ICM hypersensitivity have a 17–35% chance of reacting again upon re-exposure to HOCM, which decreases to 5% when using non-ionic compounds [52].

A recent study found that patients receiving iso-osmolar ICM had a higher incidence of adverse reactions than those receiving hypo-osmolar ICM (*p* < 0.01), while there was no difference in severe HSR [8].

Both HOCM and LOCM can induce immediate (type I) reactions with different grades of severity up to anaphylactic reactions [3, 87]. While HOCM mainly are responsible for type A reactions, low-osmolar contrast agents seem to induce a greater proportion of true allergic reactions (type B reactions) [87].

Risk status: moderate to high for type A reactions; none to low for type B reactions.

Management: apply low- or iso-osmolar ICM.

#### Mono- (low-osmolar) and dimeric (iso-osmolar) ICM

Mono- and dimeric ICMs are responsible for different types of HSR. Delayed reactions in the form of cutaneous symptoms have been shown to occur primarily after the application of dimeric and, less frequently, after monomeric ICMs (*p* < 0.05) [8, 124].

Risk status: moderate to high for dimeric compounds to induce non-immediate reactions.

Management: apply monomeric ICM in patients with dimeric ICM hypersensitivity and vice versa.

### Management- and procedure-related risk

This group of risks is the easiest to influence. The difficulty lies in identifying possible risks as such. Especially in the case of an error (see section “Risks due to an error”), which may have been or is being practiced for years, it could take a long time to identify it.

#### Risks due to an error

We identified the following five errors that may pose as risk (especially for recurrent ICM-hypersensitivity reactions):

- Incorrect assumptions regarding the culprit ICM. If an incorrect ICM is suspected as the culprit, this can have fatal consequences [137].
  Risk status: high.
  Management: exact documentation of the culprit ICM [70].
- ICM is incorrectly assumed as culprit agent. According to a previous study [138], in ~75% of cases, ICM is the trigger for HSR, and in ~25% there are other reasons (e.g., latex or skin problems occur independent of the ICM [139]). Others mention the incorrect assignment of a reaction to ICM, but they only relate this error to the so-called “physiological” reactions [7].
  Risk status: moderate to high.
  Management: search for the individual culprit factor.
- Incorrect diagnosis “iodine allergy” (see section “History of previous hypersensitivity/allergy—Iodine allergy”). Misunderstandings may arise, which also pose risks. In a one-center study, HSR following prophylactic measures was only observed in the group of patients with the diagnosis “iodine allergy” [74].
  Risk status: moderate to high.
  Management: omit the diagnosis “iodine allergy.”
- Risk of patient mix-up [140]. For example, if a patient has a contrast-HSR and it is not apparent due to confusion, a life-threatening reaction may occur.
  Risk status: moderate to high.
  Management: always make sure you are examining the right patient.
- Both over- and underestimation of possible risks are associated with hazards for the patient [141]. If prophylactic drugs are administered that are not necessary, the potential harm is greater than the benefit. If prophylaxis (not only based on a premedication) is carried out too rarely, the risk for ICM-HSR is increased.
  Risk status: moderate to high.
  Management: carry out a risk-benefit assumption in each case.

#### High injection speed

Injection speed is regularly reported as risk [8, 28, 31, 33, 60, 86]. High injection speed is associated with an increase in mild Type A reactions (e.g., heat/cold feeling, flushing, and/or headache) [1, 2]. Since patients can acquire type A and B reactions simultaneously, differentiation is not always possible. Slower injection speed reduced immediate reactions [142, 143]. Because the contrast dose was reduced simultaneously in this study, it is not clear which of these two factors contributed to the reduction in the incidence of HSR [142]. Another study clearly showed that the injection rate did not influence the prevalence of anaphylactic reactions [144].

Risk status: none for type B reactions.

Management: In patients who have responded to a high injection rate with corresponding symptoms, as prophylaxis, the lowest possible rate should be used. Injection speed does not predispose to ICM-HSR.

#### ICM dose

**Fig. 2:**
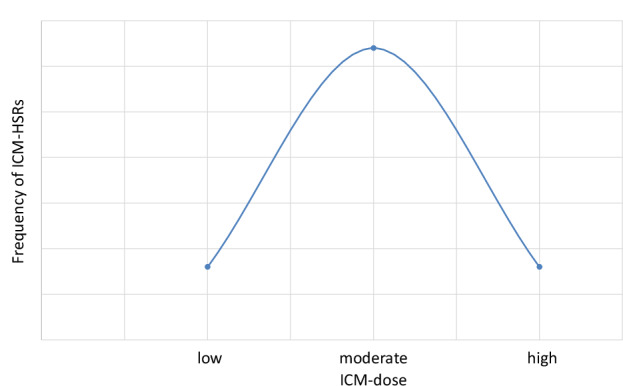
Gaussian curve demonstrating the relationship between ICM dose and frequency of ICM-hypersensitivity reactions (ICM-HSR)

A linear dose-reaction relationship is characteristic of chemotoxic (type A) reactions [52]. In HSR, there is no linear relationship between ICM dose and clinical reactivity. However, the standard doses/volumes used in CT-enhanced procedures (80–120 mL) caused most of the reactions (see sections “Intravenous ICM injection” and “Imaging modality”). Lower and higher doses are associated with lower frequencies of reactions [18, 129, 142]. This is the reason why ICM-HSR occurs rarely in arthrographic and angiographic settings [145–147]. The risk follows the shape of a Gaussian curve (Fig. 2), peaking at medium doses (< 100 mL = 45 patients; 100–200 mL = 67 patients; > 200 mL = 53 patients) [18]. Under this assumption, the use of low-dose ICM should be reassessed [129]. In addition, a lower ICM dose is associated with a more favorable CO_2_ footprint [127].

Risk status: depends on the amount of ICM applied and is greatest at the medium dose administered as part of a CECT.

Management: in patients who react to standard doses but do not react to low-dose regimens, the latter should be applied if possible.

#### First contrast medium administration

The first ICM injection in life has been classified as risk for HSR, with some patients developing anaphylaxis at first exposure [8, 10, 11, 46, 132, 148, 149]. This is problematic because all patients who receive a contrast medium for the first time in their lives should have to undergo individual prophylaxis. Neither premedication nor an allergy work-up makes sense in such patients.

Even if it looks like the first contact with a contrast medium, it may not have been the first contact. Perhaps patients have forgotten previous ICM injections, or they had a silent sensitivity due to ICM contamination in drinking water [128].

Risk status: none.

Management: none.

#### Repeated ICM application

Frequent ICM applications increase the risk of HSR [26, 30, 31, 40, 46, 51, 57, 134], affecting particularly older patients or those with chronic diseases. The cumulative incidence at the 10th, 20th, and 30th examination was 7.9%, 15.2%, and 24.1%, respectively [57]. These data show a linear dependency of the number of applied CM-doses and the incidence of HSR. However, there are no cut-off values for an increased risk. Sensitization occurs in some patients, while others remain unaffected despite multiple exposures. The more frequently contrast agents are applied, the greater the chance that sensitization will occur. However, there appears to be an unknown factor that causes sensitization to occur in certain patients and not in others. Therefore, more studies in greater patient groups are necessary to elucidate this causal connection.

Risk status: moderate.

Management: none.

#### Room temperature of the applied ICM

ICM at room temperature may cause type A reactions (e.g., heat or cold feeling, flush, pain at the injection site, headache) when applied as intravenous injection [77, 78, 150–152], especially when ionic ICM are used [150].

Discomfort can also occur following the application of non-ionic ICM at room temperature. On the other hand, HSR occurs independently of the applied ICM-temperature. The finding that extrinsic warming to 37 °C does not affect HSR rates to iopamidol [78] supports this statement. Another paper showed a reduction in the reaction rates of all types of adverse reactions [151]. The problem of this work is the unclear definition of ADR into “physiological,” “allergic-like” and global adverse reactions (whereby the sum of “physiological” and “allergic-like” reactions was greater than that of global).

Risk status: low for type A reactions.

Management: pre-warming the ICM to 37 °C in patients at risk.

#### Intravenous ICM injection

Mainly, HSR are more common following intravenous ICM injections compared to intra-arterial injections [21, 22, 42, 52, 60, 66, 79], while intra-arterial injections may cause more severe reactions [52]. Presumably, it is not the route of administration that is crucial for the occurrence of an ICM-HSR; the volume of ICM administered is the key factor in reaction rates (see section “ICM dose”).

Risk status: low to medium.

Management: none.

#### Imaging modality

CECT is one of the most often procedures in radiology. The ICM is injected intravenously. Therefore, most CM-HSR are likely to occur in the context of this procedure [39, 40, 60]. One should realize that the imaging modality is not the risk but the ICM dose (see section “ICM dose”) used.

Risk status: none.

Management: none.

#### Outpatients

Some studies report higher reaction rates in outpatients [38, 115, 121, 153], while others do not find this correlation [7]. Inpatients may have fewer documented ADRs because they leave the radiology department quickly, and reactions occurring outside the clinic are not always recorded [153]. In contrast, outpatients are more likely to have their reactions documented through the radiology staff [153]. Other authors speculated that the difficulty in accessing clinical information in outpatients, in concert with an increased number of risk factors (e.g., lack of extensive risk screening, less nursing care, and higher throughput pressure), may have contributed to this finding [5, 121]. Overall, the increased reaction frequency of outpatients seems to be a documentation deficit (ICM-HSR in inpatients occur outside of the radiology unit/clinic or risks of outpatients are missing in the Electronic health record. Therefore, this risk belongs to the group of errors (see section “Risks due to an error”).

Risk status: low.

Management: improve documentation of ICM-related HSR.

## Conclusion and outlook

Despite numerous publications, the predictability of ICM-hypersensitivity reactions remains limited. Our analysis shows that many reported risk factors are inconsistent, overlapping, or not clinically relevant. Critically, only four factors should be emphasized: a history of ICM hypersensitivity, acute allergy, the culprit ICM and previously not identified documentation errors. These risks directly affect patient safety and highlight the need for precise identification and consistent reporting, rather than an uncritical accumulation of nonspecific factors.

Interestingly, there is sometimes information in the literature that precautionary measures should be taken in high-risk patients, but there is no explanation of what risk is meant (e.g., for renal insufficiency, a hypersensitivity reaction). Such information is not helpful but rather confusing. Also confusing is the fact that the current literature dealing with risk factors gives no information about prophylaxis.

Sometimes one has the impression that risk factors are listed uncritically or that the underlying mechanisms are unclear to the authors (e.g., that chronically ill patients often have to undergo contrast-enhanced imaging and therefore the disease itself does not represent the risk). Moreover, if one does not understand the pathology of ICM-hypersensitivity reactions, it is not possible to define risks. In the future, intensive training will be necessary, which will ultimately serve to ensure patient safety.

## Supplementary information

ELECTRONIC SUPPLEMENTARY MATERIAL

## Data Availability

Data supporting the results reported in the article can be found in the publications listed under “References.” Other data were not used.

## Abbreviations

ACE: Angiotensin converting enzyme
ADR: Adverse drug reaction(s)
BMI: Body mass index
CECT: Contrast-enhanced computed tomography
CI-AKI: Contrast-induced acute kidney injury
CM: Contrast medium/media
CT: Computed tomography
HOCM: High-osmolar contrast medium/media
HSR: Hypersensitivity reaction(s)
ICM: Iodinated contrast medium/media
IHR: Immediate hypersensitivity reaction(s)
IL-2: Interleukin-2
LOCM: Low-osmolar contrast medium/media
mL: Millilitres
NIHR: Non-immediate hypersensitivity reaction(s)
OR: Odds ratio(s)
