# Supplementary material for: Risk factor analysis of iodinated contrast medium-related hypersensitivity reactions
Source: Insights Imaging. 2025 Oct 10;16:216. doi: 10.1186/s13244-025-02099-y (PMC12514124; doi:10.1186/s13244-025-02099-y)
Supplement: Supplementary file 1 — ELECTRONIC SUPPLEMENTARY MATERIAL [file 13244_2025_2099_MOESM1_ESM.docx]

**Supplement 1**

Odds ratio analysis on the basis of the following papers [5,7,10,17,18,21,22,26,34, 38,44,58,82,86,88,104,109,117,118,119,121,122,132,133].

**1. Patient-related Risk Factors**

A history of previous hypersensitivity or allergic reaction to iodinated contrast media (ICM) emerged as the strongest predictor of future hazard ratio (HR), with odds ratios ranging from 2.0 to 27.6 across multiple studies [5,7,10,17,18,21,22,26,44,86,109,132]. Other allergic conditions, such as drug allergy (ORs: 1.9–5.7, p < 0.05) [18,118,132], allergic rhinitis (OR: 1.9-2.3, p<0.05) [10,18], asthma bronchiale (ORs: 1.1–8.7) and food allergy (1.5-7.8) were also associated with increased risk [7,10,17,18, 26,86,117]. Pollen seasons, considered a surrogate for acute allergy status, also presented elevated risk with OR of 2.8 [5,34].

Systemic diseases such as systemic lupus erythematosus (OR: 1.4 [82]), cancer (OR: 1.9 [26]), and mastocytosis were identified as predisposing conditions. Diabetes mellitus and hypertension were generally associated with slightly increased, but non-significant, odds ratios (Diabetes mellitus OR: 1.0–1.5; hypertension OR: 1.0–1.1 [5,22,26,86]). Cardiovascular disease showed mixed results, with odds ratios ranging from 0.6 to 2.2 [10,44,86,88].

Female sex was associated with a significantly higher risk of HRS (e.g., OR: 2.4, [38,119]), as was younger age, particularly in the 20–40 year group (e.g., OR: 2.3 [7]). Motosugi et al presented an odds ratio of 0.98 per 1-year decrease in age, *p* = 0.0019, meaning that your patients are in risk [122]. Ethnic background was also a contributing factor, with both White (OR: 1.8, 95% CI: 1.7–1.9, p < 0.01) and Non-White individuals (OR: 1.8, 95% CI: 1.4–2.3, p < 0.01) demonstrating elevated risks [7,17,117].

**2. ICM-related Risk Factors**

The type of ICM compound had a influence on reaction risk. Iomeprol (OR: 6.8 [58]) and iopromide (OR: 3.1 [7]) were among the agents with higher associated risks. Osmolality and molecular structure (e.g., monomer vs. dimer) also played a role, with higher osmolality contrast agents generally associated with greater risk (OR: 2.2 [44]). Culprit and cross-reactive ICMs were repeatedly implicated in recurrent reactions.

**3. Management-/Procedure-related Risk Factors**

Errors in diagnosis or documentation, such as misattributed "iodine allergy," were frequent contributors to inappropriate management. Incorrect assumptions regarding the culprit ICM or its risk potential were also identified as procedural pitfalls.

Among procedural factors, high injection speed (OR: 1.8 [86]) and first-time ICM administration (OR: 2.2 [10]) were linked to elevated risk. Compared to <20 g of iodine dose, slightly increased odds ratios were observed across higher dose groups. Significant associations were found for 20–40 g (OR 1.3, 95% CI: 1.0–1.6, p = 0.04) and >60 g iodine dose (OR 1.4, 95% CI: 1.1–1.7, p = 0.01) [21].

Repeated ICM injections or a history of prior ICM exposure were associated with increased risk, with odds ratios of 2.4 (95% CI: 1.3–4.8, [104]) and 2.9 (95% CI: 1.2–7.0, p = 0.02, [133]). Intravenous administration showed a significant effect compared to other administration routes (e.g. intra-arterial) (OR: 3.6 95% CI 2.8–4.8, p < 0.01 [21,22]). Additionally, certain procedural settings such as outpatient imaging (e.g., OR: 7.5 [121]) were associated with significantly increased risk.

**Supplement 2**

**Table**

Risk factors mentioned in the literature that could predispose to ICM hypersensitivity reactions including the corresponding odds ratios (ACE – angiotensin-converting enzyme; BMI – body mass index; CECT – contrast-enhanced computed tomography; HOCM – high-osmolal contrast medium; HR – hazard ratio; ICM – iodinated contrast media; IR – immediate reaction; LOCM – low-osmolal contrast medium; NIR - non-immediate reaction).

| **Risk factor** | **References (first author, year, [reference])** | **Odds ratio (95%-interval, p-value) or HR** |
| --- | --- | --- |
| **Patient-related risks** | | |
| Acute allergy | Bettmann 1997 [17], Chen 2021 [18], Chiu 2022 [19], Costa 2004 [20], Endrikat 2020 [21], Endrikat 2022 [22], Endrikat 2024 [23], Guéant-Rodriguez 2006 [24], Kim 2014 [11], Kobayashi 2012 [25], Lee 2019 [26], Morzycki 2016 [27], Newmark 2012 [28], Palkowitsch 2014 [29], Palmiere 2014 [30], Rosado-Ingelmo 2016 [31], Schopp 2013 [32], Singh 2008 [33], Vogl 2006 [34], Voltolini 2022 [10], Wendt 2006 [35], Wolf 1989 [36] |  |
| Pollen seasons | Bellin 2011 [37], Boehm 2009 [4], Ho 2012 [38]^H^, Kim 2012 [39], Munechika 2003 [40], Wolf 1989 [36], Zeng 2024 [5]^Z^ | 2.8 (1.4-5.6, <0.01)^H^  1.6 (1.0-1.9, <0.01)^Z^ |
| History of hypersensitivity / allergy to ICM | Aggarwal 2015 [109]^A^, Andreucci 2014 [43], Barrett 1992 [44]^Ba^, Bettmann 1997 [17]^Be^, Beiner 2024 [45], Bellin 2011 [37], Biló 2022 [46], Bottinor 2013 [47], Brockow 2005 [48], Brockow 2009 [49], Brockow 2022 [50], Brockow 2014 [51], Bush 1991 [52], Cha 2019 [53]^Cha^, Chen 2021 [18]^Che^, Chiu 2022 [19], Cochran 2005 [54], Costa 2004 [20], Cruz 2022 [55], Dellis 2022 [56], Endrikat 2020 [21]^E1^, Endrikat 2022 [22]^E2^, Endrikat 2024 [23], Fujiwara 2013 [57], Fukushima 2023 [58], Guéant-Rodriguez 2006 [24], Idée 2005 [42], Jenerowicz 2022 [59], Katayama 1990 [12], Kim 2012 [39], Kim 2014 [11], Kobayashi 2013 [133]^K^, Kopp 2008 [60], Lee 2019 [26]^L^, Li 2017 [8], McDonald 2023 [7]^Mc^, Morcos 2008 [61], Morzycki 2016 [27], Munechika 2003 [40], Namasivayam 2006 [62], Newmark 2012 [28], Palkowitsch 2014 [29], Palmiere 2014 [30], Park 2017 [63], Petersein 2011 [64], Pradubpongsa 2013 [65], Rosado-Ingelmo 2016 [31], Schopp 2013 [32], Shehadi 1975 [66], Vogl 2006 [34], Voltolini 2022 [10]^V^, Wendt 2006 [35], Wolf 1989 [36], Zeng 2024 [5], Zhang 2014 [86]^Zh^ | 3.7 (1.5-9.3, 0.02)^A^  1.8 (1.2-2.7, -)^Ba^  2.0 (-, <0.05)^Be^  199 (49-802, <0.001)^Cha^ 2.4 (1.1-4.3, 0.04)^Che^  4.3 (2.8-6.8, <0.01)^E1^  4.3 (2.7-6.7, <0.01)^E2^  7.1 (5.2-9.7, <0.01)^K^  HR: 40.7 (35.5-46.7, <0.01)^L^  27.6 (22.4-34.0 <0.01)^Mc^  2.0 (1.1-3.8, 0.03)^V^  10.2 (2.1-49.9, <0.01)^Zh^ |
| History of iodine allergy | Newmark 2012 [28], Scherer 2010 [73], Trautmann 2019 [72] |  |
| Family history of ICM-hypersensitivity | Brockow 2022 [50], Cha 2019 [53]^C^ | 14 (2-112, 0.01)^C^ |
| History of allergy (drug allergy, atopy, asthma bronchiale, allergic rhinitis, food allergy) | Andreucci 2014 [43], Ansell 1980 [13], Barrett 1992 [44]^Ba^, Bettmann 1997 [17]^Be^, Bellin 2011 [37], Biló 2022 [46], Bottinor 2013 [47], Brockow 2005 [48], Brockow 2009 [49], Brockow 2014 [51], Brockow 2022 [50], Bush 1991 [52], Callahan 2009 [41], Cha 2019 [53]^Cha^, Chen 2021 [18]^Che^, Cochran 2005 [54], Costa 2004 [20], Davenport 2012 [78], Dellis 2022 [56], Endrikat 2020 [21]^E1^, Endrikat 2022 [22]^E2^, Fujiwara 2013 [57], Guéant-Rodriguez 2006 [24], Goksel 2011 [117]^G^, Idée 2005 [42], Jenerowicz 2022 [59], Katayama 1990 [12], Kim 2012 [39], Kobayashi 2013 [133]^K^, Lang 1993 [88]^La^, Lee 2019 [26]^Le^, Li 2017 [8], McDonald 2023 [7]^Mc^, Morcos 2008 [61], Morzycki 2016 [27], Munechika 2003 [40], Namasivayam 2006 [62], Newmark 2012 [28], Palkowitsch 2014 [29], Palmiere 2014 [30], Park 2017 [63], Petersein 2011 [64], Pradubpongsa 2013 [65], Schopp 2013 [32], Shehadi 1975 [66], Singh 2008 [33], Smith 2019 [79], Thomas 1999 [80], Torres 2012 [81], Vogl 2006 [34], Voltolini 2022 [10]^V^, Wendt 2006 [35], Wolf 1989 [36], Zeng 2024 [5], Zhang 2014 [86]^Zh^ | Allergy:  1.6 (1.2-2.0, -)^Ba^  1.7 (-, <0.05)^Be^  6.8 (3.2-14.8, 0.01)^Cha^  3.6 (2.8-4.6, <0.01)^E1^  3.7 (2.9-4.7, <0.01)^E2^  1.2 (1.0-1.4, 0.02)^Mc^  Drug allergy:  1.9 (0.9-4.0, 0.09)^Cha^  2.5 (1.12-4.2, 0.02)^Che^  1.9 (1.5-2.6, <0.01)^K^  5.7 (2.3-14.4, <0.01)^G^  Allergic rhinitis:  1.9 (1.1-2.9, 0.02)^Che^  HR: 1.5 (1.0-2.2, -)^Le^  2.3 (1.1-4.8, 0.03)^V^  Food allergy:  1.5 (0.1-1.6, 0.62)^Che^  7.8 (1.2-52.0, <0.05)^G^  1.5 (0.4-5.5, 0.56)^V^  Asthma:  1.1 (-, >0.05)^Be^  3.5 (1.9-6.6, 0.01)^Cha^  1.5 (1.1-2.8, 0.02)^Che^  4.4 (1.8-10.7, <0.01)^G^  8.7 (2.4-32.3, <0.01)^La^  HR: 1.5 (1.1-2.0, -)^Le^  1.1 (0.9-1.3, 0.21)^Mc^  1.4 (0.2-10.3, 0.75)^Zh^ |
| **Serious / chronic disease** |  |  |
| Cancer | Biló 2022 [46], Dellis 2022 [56], Lee 2019 [26]^L^, Kim 2012 [39], Rosado-Ingelmo 2016 [31] | HR: 1.9 (1.7-2.1, <0.01)^L^ |
| Systemic lupus erythematosus | Bellin 2011 [37], Bottinor 2013 [47], Brockow 2005 [48], Guéant-Rodriguez 2006 [24], Jenerowicz 2022 [59], Morzycki 2016 [27], Rawal 2022 [82]^R^ | 1.4 (1.4-1.5, <0.01)^R^ |
| Mastocytosis | Bonadona 2014 [90], Brockow 2005 [48], Brockow 2009 [49], Brockow 2011 [91], Carter 2019 [92], Gianetti 2014 [93], Hermans 2017 [94], Jenerowicz 2022 [59], Morzycki 2016 [27], Newmark 2012 [28], Turner 2017 [95], Voltolini 2022 [10] |  |
| Renal complaints | Barrett 1992 [44], Bellin 2011 [37], Brockow 2014 [51], Chen 2021 [18], Costa 2004 [20], Fujiwara 2013 [57], Kim 2012 [39], Lee 2019 [26]^L^, Li 2017 [8], Modi 2012 [85], Morzycki 2016 [27], Namasivayam 2006 [62], Palmiere 2014 [30], Petersein 2011 [64], Rosado-Ingelmo 2016 [31], Singh 2008 [33], Vogl 2006 [34], Wendt 2006 [35], Wolf 1989 [36], Zeng 2024 [5] | HR: 0.7 (0.5-1.2, 0.25)^L^ |
| BMI / body weight | Li 2017 [8], McDonalds 2023 [7]^Mc^, Kim 2017 [118], Vogl 2006 [34], Zhang 2014 [86]^Zh^ | 1.0 (0.9-1.1, 0.70)^Mc^  1.0 (1.0-1.0, <0.01)^K^  1.7 (1.2-2.4, <0.01)^Zh^ |
| Others (e.g. anemia, cardiovascular disease, diabetes mellitus, gout, hepatic disease) | Barrett 1992 [44]^B^, Biló 2022 [46], Chiu 2022 [19], Endrikat 2022 [22]^E^, Idée 2005 [42], Katayama 1990 [87], Katayama 1990 [12], Kim 2012 [39], Lang 1993 [88]^La^, Lee 2019 [26]^L^, Li 2017 [8], Rosado-Ingelmo 2016 [31], Schopp 2013 [32], Singh 2008 [33], Thomas 1999 [80], Voltolini 2022 [10]^V^, Wolf 1989 [36], Zeng 2024 [5]^Ze^, Zhang 2014 [86]^Zh^ | Hypertension:  1.1 (0.9-1.4, <0.01)^E^  HR: 0.7 (0.7-1.0, 0.06)^L^  1.0 (0.3-3.3, 0.99)^Ze^  1.0 (0.7-1.5, 0.87)^Zh^  Diabetes mellitus:  1.5 (1.2-2.0, <0.01)^E^  HR: 1.1 (0.9-1.3, 0.09)^L^  1.7 (0.5-5.5, 0.4)^Ze^  1.0 (0.6-1.5, 0.87)^Zh^  Chronic liver disease:  HR: 2.1 (1.8-2.4, <0.01)^L^  Cardiovascular disorder:  1.4 (1.1-1.9, -)^B^  2.2 (1.0-4.9, 0.05)^La^  2.1 (1.3-3.4, <0.01)^V^  0.6 (0.2-2.6, 0.53)^Zh^  Concomitant disease:  1.4 (1.2-1.7, <0.01)^E^ |
| Patient’s anxiety | Barrett 1992 [44], Chiu 2022 [19], Goksel 2011 [117]^G^, Idée 2005 [42], Kim 2012 [39], Liccardi 2008 [97], Newmark 2012 [28] | Psychiatric disease:  6.4 (2.1-19.1, <0.01)^G^ |
| The way of talking to patients | Hopper 1994 [100], Spring 1984 [101], Yucel 2005 [102] |  |
| **Medication** |  |  |
| Beta-blockers | Aggarwal 2015 [109]^A^, Andreucci 2014 [43]^A^, Jenerowicz 2022 [59], Kim 2012 [39], Lang 1991 [105], Lang 1993 [96]^L^, Palmiere 2014 [30], Rosado-Ingelmo 2016 [31], Singh 2008 [33] | 1.1 (0.5-2.2, 0.84)^A^  1.8 (0.8-4.3, 0.18)^L^ |
| Interleukin-2 | Brockow 2014 [51], Choyke 1992 [103], Drljevic-Nielsen 2023 [111], Idée 2005 [42], Jenerowicz 2022 [59], Kim 2012 [39], Morcos 2008 [61], Newmark 2012 [28], Oldham 1990 [107], Rosado-Ingelmo 2016 [31], Singh 2008 [33], Zukiwski 1990 [108] |  |
| ACE inhibitors | Rosado-Ingelmo 2016 [31], Voltolini 2022 [10]^V^ | 1.4 (0.8-2.6, 0.25)^V^ |
| Taxanes | Farolfi 2014 [104]^Fa^ | 2.1 (1.0-4.2, -)^F^ |
| Anti-CTLA4 antibodies  Other drugs | Ridolfi 2018 [106]  Munechika 2003 [40] |  |
| **Gender** |  |  |
| Female | An 2019 [114], Bellin 2011 [37], Bettmann 1997 [17], Brockow 2014 [51], Chen 2017 [115], Deng 2019 [116]^D^, Endrikat 2020 [21], Endrikat 2022 [22], Endrikat 2024 [23], Farolfi 2014 [104]^Fa^, Fukushima 2023 [58]^Fu^, Goksel 2011 [117]^G^, Ho 2012 [38]^H^, Idée 2005 [42], Jenerowicz 2022 [59], Kim 2017 [118], Kopp 2008 [60], Lang 1995 [119]^La^, Lee 2019 [26], Li 2017 [8], Maurer 2010 [120], McDonald 2023 [7]^Mc^, Mortelé 2005 [121]^M^, Munechika 2003 [40], Newmark 2012 [28], Palkowitsch 2014 [29], Pradupbongsa 2013 [65], Rosado-Ingelmo 2016 [31], Singh 2008 [33], Vogl 2006 [34], Voltolini 2022 [10]^V^, Wang 2008 [123], Wendt 2006 [35], Zeng 2024 [5], Zhang 2014 [86] | 1.7 (1.7-1.7 <0.01)^D^  1.2 (1.0-1.3, 0.03)^E1^  1.2 (1.0-1.3, 0.03)^E2^  1.2 (0.8-1.9, -)^Fa^  1.0 (0.5-1.8, 0.90)^Fu^  2.2 (1.0-3.9, <0.05)^G^  2.4 (1.3-4.6, <0.01)^H^  1.5 (1.4-1.7, <0.01)^K^  2.4 (1.4-4.1, <0.01)^La^  1.3 (1.1-1.4, <0.01)^Le^  3.1 (1.5-7.0, <0.05)^M^  1.5 (1.3-1.7, <0.01)^Mc^  1.8 (1.3-2.7, <0.01)^V^ |
| Male | Chen 2021 [18]^C^, Motosugi 2016 [122], Thomas 1999 [80] | 1.2 (0.8-1.8, 0.69)^C^ |
| **Age** | Motosugi 2016 [122]^M^ | 0.98 per 1-year increase in age, *p* = 0.0019^M^ |
| < 20 years | Kim 2012 [39], Singh 2008 [33], Vogl 2006 [34] |  |
| 20 – 40 years | Bush 1991 [52], Endrikat 2020 [21]^E^, Endrikat 2022 [22], Fukushima 2023 [58]^F^, Gomi 2010 [132], Katayama 1990 [87], Kim 2017 [118]^K^, Kopp 2008 [60], Li 2017 [8], McDonald 2023 [7]^Mc^, Palkowitsch 2014 [29], Petersein 2011 [64], Schopp 2013 [32], Shehadi 1975 [66], Singh 2008 [33], Sohn 2019 [134], Vogl 2006 [34] | 2.16 (1.78-2.62, <0.01)^E^  2.1 (0.8-5.4, 0.14)^F^  1.5 (1.0-2.4, 0.04)^K^  2.3 (1.7-3.0, <0.01)^Mc^ |
| 41 – 60 years | An 2019 [114], Biló 2022 [46], Endrikat 2020 [21]^E1^, Endrikat 2022 [22], Farolfi 2014 [104]^F^, Ho 2012 [38]^H^, Kim 2017 [118], Kobayashi 2013 [133]^K^, Lee 2019 [26]^L^, Li 2017 [8], McDonald 2023 [7], Palkowitsch 2014 [29], Schopp 2013 [32], Sohn 2019 (IR) [134], Voltolini 2022 [10]^V^, Zhang 2014 [86] | 2.5 (1.3-4.8, <0.01)^E^  1.7 (1.1-2.6, -)^F^  1.8 (1.2-2.6, <0.01)^H^  1.8 (1.4-2.2, <0.01)^K^  HR: 2.1 (1.9-2.3, <0.01)^L^ 1.6 (1.1-2.4, 0.01)^V^ |
| > 60 years | Biló 2022 [46], Farolfi 2014 [104], Kim 2012 [39], Kopp 2008 [60], Singh 2008 [33], Sohn 2019 (NIR) [134], Voltolini 2022 [10], Zhang 2014 [86] |  |
| Ethnic background | Ansell 1980 [13], Bettmann 1997 [17]^B^, Deng 2019 [116]^D^, Endrikat 2020 [21], Endrikat 2024 [23], Idée 2005 [42], Jenerowicz 2022 [59], McDonald 2023 [7]^Mc^ | White:  0.8 (-,-)^B^  1.8 (1.7-1.9, <0.01)^D^  Non-White:  1.8 (1.4-2.3, <0.01)^Mc^ |
| **ICM-related risks** | | |
| ICM compound (e.g. iopromide, iodixanol) | An 2019 [114], Chen 2017 [115], Chen 2021 [18]^C^, Fukushima 2023 [58]^F^, Gomi 2010 [132], Kim 2017 [118]^K^, Lee 2019 [26], McDonalds 2023 [7]^Mc^, Motosugi 2016 [122]^M^, Sauer 2022 [135], Sohn 2019 [134]^S^, Zeng 2024 [5] | Iohexol:  2.0 (1.2-4.0, 0.03)^C^  1.4 (1.1-1.7, <0.01)^K^  Iodixanol:  1.6 (1.1-2.4, 0.02)^S^  1.8 (0.7-3.3, 0.09)^C^  1.0 (0.6-1.6, 0.98)^Mc^  Iomeprol:  6.8 (2.8-16.4, <0.01)^F^  1.9 (1.3-2.9, <0.01)^M^ Iopromide:  2.3 (0.7-16.6, 0.12)^F^  2.7 (2.2-3.4, <0.01)^K^  3.1 (2.4-4.0, <0.01)^Mc^  Iopamidol:  1.6 (1.3-2.0, <0.01)^K^  1.0 (0.6-1.5, 0.87)^M^  Ioversol:  0.9 (0.5-1.6, 0.67)^M^ |
| Culprit ICM | Abe 2016 [76], Beiner 2024 [45], Katayama 1990 [87] |  |
| Cross-reacting ICM | Ahn 2014 [125], Brockow 2009 [49], Torres 2012 [81], Trautmann 2019 [72] |  |
| Osmolality (HOCM > LOCM > IsoOCM) | Abe 2016 [76], Barrett 1992 [44]^B^, Bettmann 1997 [17], Cochran 2000 [54], Federle 1998 [144], Katayama 1990 [87], Li 2017 [8], Wolf 1989 [36] | 2.2 (1.6-2.9, -)^B^ |
| Mono- versus dimeric ICM | Schild 2006 [124], Li 2017 [8] |  |
| **Management- / procedure-related risks** | | |
| Incorrect assumption regarding the culprit ICM | Zuckermann 1998 [137] |  |
| ICM is incorrectly assumed to be the culprit agent | Böhm 2011 [138], Böhm 2022 [139], McDonald 2023 [7] |  |
| Incorrect documentation and incorrect diagnosis such as “iodine allergy” | Böhm 2017 [74], Deng 2019 [116]^D^ |  |
| Patient mix-up | Yoshimura 2003 [140] |  |
| Over- and underestimation of risks | Lombardo 2023 [141] |  |
| Outpatient | Chen 2017 [115], Dean 2015 [153], Fukushima 2023 [58]^F^, Ho 2012 [38]^H^, Mortelé 2005 [121]^M^ | 1.8 (0.6-5.2, 0.27)^F^  5.4 (2.8-10.8, <0.01)^H^  7.5 (4.3-11.2 <0.05)^M^ |
| High injection speed | Barrett 1992 [44]^B^, Federle 1998 [144], Kopp 2008 [60], Li 2017 [8], Newmark 2012 [28], Rosado-Ingelmo 2016 [31], Singh 2008 [33], Zhang 2014 [86]^Zh^ | 1.8 (1.4-2.5, <0.01)^Zh^ |
| ICM dose | Barrett 1992 [44], Endrikat 2020 [21]^E^, Kobayashi 2013 [133]^K^, Li 2017 [8], McDonald 2023 [7]^Mc^, Mortelé 2005 [121]^M^ | 1.3 (1.1-1.7, 0.02)^Mc^  >20–40g:  1.3 (1.0-1.6, 0.04)^E^  >40–60g:  1.2 (1.0-1.5, 0.07)^E^  1.3 (0.8-2.1, > 0.05)^M^  >60 g:  1.3 (0.7-2.3, 0.37)^E^  1.4 (1.1-1.7, 0.01)^K^ |
| First ICM-injection | Biló 2022 [46], Gomi 2010 [132], Hasdenteufel 2008 [149], Hosoya 2000 [148], Li 2017 [8], Voltolini 2022 [10]^V^ | 2.2 (1.4-3.5, <0.01)^V^ |
| Repeated ICM-injection or history of prior ICM-exposure | Biló 2022 [46], Brockow 2014 [51], Farolfi 2014 [104]^Fa^, Fujiwara 2013 [57], Lee 2019 [26], Munechika 2003 [40], Palmiere 2014 [30], Rosado-Ingelmo 2016 [31], Sohn 2019 [134]^S^ | 2.4 (1.3-4.8, -)^Fa^ 2.9 (1.2-7.0, 0.02)^S^ |
| ICM at room temperature | Basharat 2022 [77], Davenport 2012 [78], Nawras 2023 [152], Vergara 1996 [150], Zhang 2018 [151] |  |
| Intravenous ICM-injection | Endrikat 2020 [21]^E1^, Endrikat 2022 [22]^E2^, Idée 2005 [42], Kopp 2008 [60], Shehadi 1975 [66], Smith 2019 [79] | 3.6 (2.8-4.8, <0.01)^E1^  3.6 (2.8-4.8, <0.01)^E2^ |
| Imaging modality (CECT)  Invasive procedure | Kim 2012 [39], Kopp 2008 [60], Munechika 2003 [40], Zhang 2014 [86]  Munechika 2003 [40] |  |
| Hydration after radiography | Munechika 2003 [40], Motosugi 2016 [122] |  |
